# Decreased PGC1β expression results in disrupted human erythroid differentiation, impaired hemoglobinization and cell cycle exit

**DOI:** 10.1038/s41598-021-96585-0

**Published:** 2021-08-24

**Authors:** Taha Sen, Jun Chen, Sofie Singbrant

**Affiliations:** Division of Molecular Medicine and Gene Therapy, Lund Stem Cell Center, Lund University, Lund, Sweden

**Keywords:** Haematopoietic stem cells, Stem-cell differentiation, Differentiation, Erythropoiesis, Cell division, Cell-cycle exit, Anaemia, Myelodysplastic syndrome, Mitochondria

## Abstract

Production of red blood cells relies on proper mitochondrial function, both for their increased energy demands during differentiation and for proper heme and iron homeostasis. Mutations in genes regulating mitochondrial function have been reported in patients with anemia, yet their pathophysiological role often remains unclear. PGC1β is a critical coactivator of mitochondrial biogenesis, with increased expression during terminal erythroid differentiation. The role of PGC1β has however mainly been studied in skeletal muscle, adipose and hepatic tissues, and its function in erythropoiesis remains largely unknown. Here we show that perturbed PGC1β expression in human hematopoietic stem/progenitor cells from both bone marrow and cord blood results in impaired formation of early erythroid progenitors and delayed terminal erythroid differentiation in vitro, with accumulations of polychromatic erythroblasts, similar to MDS-related refractory anemia. Reduced levels of PGC1β resulted in deregulated expression of iron, heme and globin related genes in polychromatic erythroblasts, and reduced hemoglobin content in the more mature bone marrow derived reticulocytes. Furthermore, PGC1β knock-down resulted in disturbed cell cycle exit with accumulation of erythroblasts in S-phase and enhanced expression of G1-S regulating genes, with smaller reticulocytes as a result. Taken together, we demonstrate that PGC1β is directly involved in production of hemoglobin and regulation of G1-S transition and is ultimately required for proper terminal erythroid differentiation.

## Introduction

Mammalian red blood cells (RBC) are the most abundant cells in the body, with the primary function to transport oxygen and clear distal tissues of carbon dioxide. RBCs have a finite lifetime of approximately 120 days^1^ and require continuous production through erythropoiesis, a step-wise process requiring delicate coordination of intrinsic and extrinsic regulators^2^. Imbalanced coordination drives the pathogenesis of various anemia related disorders. During erythropoiesis pluripotent hematopoietic stem cells differentiate through a series of tightly regulated intermediate steps into mature RBC^2^. The first erythroid committed colony forming progenitors BFU-E’s and the subsequent CFU-E’s have activated expression of the receptor for erythropoietin (EPO)^3^, the main hormone responsible for maintenance and proliferation of erythroid committed progenitors during definitive erythropoiesis^4,5^. During the subsequent terminal differentiation erythroblasts undergo stepwise maturation, becoming smaller and increasingly hemoglobinized with each division, condense their nuclei, autophagocytize mitochondria and other organelles, and finally exit cell cycle and extrude their nuclei^6,7^.

Mitochondrial biogenesis is enhanced through post-transcriptional modifications in human erythropoiesis^8^, and recent advances have implicated that mitochondrial dysfunction leads to impaired erythropoiesis^8–15^, indicating that proper mitochondrial function is critical for erythroid development. Differentiating erythroblasts have increased energy demands, which mitochondria supply by ramping up oxidative phosphorylation (OXPHOS). Additionally, mitochondria are responsible for heme and iron metabolism, two components critical for proper function of erythrocytes. The PPAR-gamma coactivator-1 (PGC-1) family of transcriptional coactivators have been shown to regulate mitochondrial capacity and energy metabolism through various nuclear receptors and recruitment of protein complexes to specific DNA sequences^16^. They are preferentially expressed in tissues with high OXPHOS needs, and the first member, PGC1α was identified in brown adipose tissue where it was shown to regulate mitochondrial biogenesis^17,18^. The PGC1α homologs, PGC1β and PGC-1-related coactivator (PRC), exert similar downstream effects^19,20^. However, due to tissue-specific expression patterns, the various PGC1 coactivators have distinct physiological roles dependent on the availability of the numerous nuclear receptors through which cellular metabolism is controlled^16^.

Pgc-1β display increased expression during terminal erythroid differentiation^21^. However, its function has mainly been studied in adipocytes, hepatic-, cardiac and skeletal tissues^19,22–25^, while the role of PGC-1β in erythropoiesis remains largely unknown. The only study to date directly evaluating the role of PGC-1 coactivators during RBC development demonstrates that compound deletion of the related *Pgc1*α and *Pgc1*β during embryonic development in mouse results in anemia due to deregulation of globin genes^26^. Furthermore, we recently demonstrated that perturbed PGC1β expression in response to pRb-deletion in mice results in a developmental block during terminal erythropoiesis and anemia reminiscent of that reported in Myelodysplastic syndrome (MDS)^21,27^. Importantly, the MDS-like anemia could be rescued by enhanced PPAR-signaling, either through PGC1β over-expression or Bezafibrate treatment^21^, indicating that PGC1β plays a functional role in the anemic phenotype. Notably, PGC1β is haplo-insufficient in the chromosome 5q deletion subtype of MDS (del(5q) MDS)^28^ and deregulation of core mitochondrial pathways and acquired mtDNA mutations have been reported in MDS-patients with anemia^29^, generally in genes related to iron and heme homeostasis^12,30,31^. However, the role of PGC1β in regulation of human erythropoiesis is yet to be determined.

To decipher the role of PGC1β in human erythropoiesis, PGC1β was knocked down in bone marrow derived CD34^+^ hematopoietic stem/progenitor cells and investigated for its effects during erythroid differentiation. Here we show that PGC1β is required for proper human erythroid development, and that perturbed expression results in delayed differentiation in vitro, with accumulations of polychromatic erythroblasts similar to what is seen in MDS-related refractory anemia. Transcriptional and functional analysis revealed that reduced levels of PGC1β resulted in perturbed hemoglobin production, as well as disturbed cell cycle exit with smaller reticulocytes as a result.

### Results

**Figure 1:**
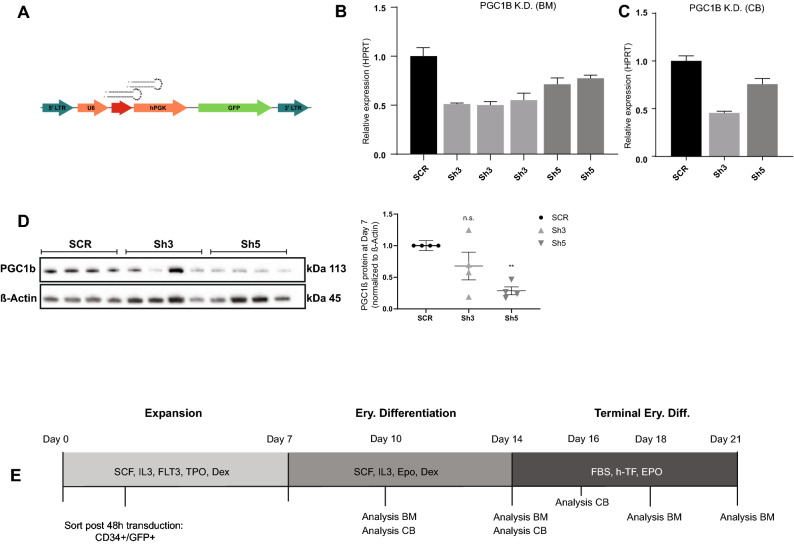
Experimental setup to study perturbed PGC1β signaling during human erythroid development. (**A**) Description of the pLKO lentiviral vector used, expressing a scrambled control vector (Scr) or two different short hairpin RNA for PGC1β, sh3 and sh5. Initial transduction efficiency was on average 65% on day3 (SCR: 62%, sh3: 66%, sh5 66%). (**B**) Quantification of knock-down efficiency in transduced cells at the transcriptional level in B) bone marrow derived CD34 + cells and (**C**) cord blood derived CD34 + cells. (n = SCR:4, Sh3:12, Sh5:6 for BM and n = 4 for CB). (**D**) Knock-down efficiency of PGCβ at the protein level in cord blood derived progenitors on day7 as assessed by western blot. Quantification of PGC1β protein was normalized to ß-ACTIN. (**E**) Schematic outline of the 3-phase erythroid culturing system of human CD34^+^ cells (modified from Hu et al. 2013)^32^. 25,000 transduced CD34 + BM cells or 100,000 transduced CD34 + CB cells were seeded on day 3, and split on days of medium switching, with average cell concentrations of 9 × 10^5^ cells/ml, 3.5 × 10^6^ cells/ml and 2 × 10^5^ cells/ml on days 14, 18 and 21 respectively. Data is presented as mean ± SEM (*P ≤ 0.05, **P ≤ 0.01, ***P ≤ 0.001).

*Pgc1* coactivators have been indicated to play a role during murine erythropoiesis^26^, while their function in human erythropoiesis remains unknown. To further decipher the role of PGC1β during human erythroid development, we reduced the PGC1β expression in human CD34^+^ bone marrow (BM) and cord blood (CB) progenitors using lentiviral knock-down with two different shRNAs (sh3, sh5) to reduce the risk of off-target effects (Fig. 1A), and investigated its effects during erythroid differentiation in vitro. High initial transduction efficiency, on average 65%, was achieved with both vectors (data not shown), with sh3 consequently resulting in more efficient knock-down of PGC1β expression than sh5, averaging 48% and 26% respectively for BM (Fig. 1B, n = 4 biological replicates times 3 and 2 separate transductions for sh3 and sh5 respectively), and 55% and 25% respectively for CB (Fig. 1C). PGC1β knock-down efficiency was further assessed at the protein level in CB derived CD34^+^ cells. Surprisingly, sh5 with less efficient mRNA knock-down at the transcriptional level displayed a more effective reduction at the protein level (Fig. 1D, full blot in Fig. S1). Transduced (GFP^+^) CD34^+^ BM and CB cells were sorted and the effects of perturbed PGC1β expression were analyzed using a three-phase culture system that over 21 days effectively recapitulates human erythroid development from hematopoietic stem/progenitor cells to reticulocytes, including all intermediate erythroid precursors (Fig. 1E, modified from Hu et al. 2013)^32^, with some differences in kinetics in proliferation and differentiation between CD34^+^ progenitors derived from fetal and adult sources ^33^.

### Decreased expression of PGC1β results in perturbed formation of early erythroid progenitors and delayed terminal differentiation

**Figure 2:**
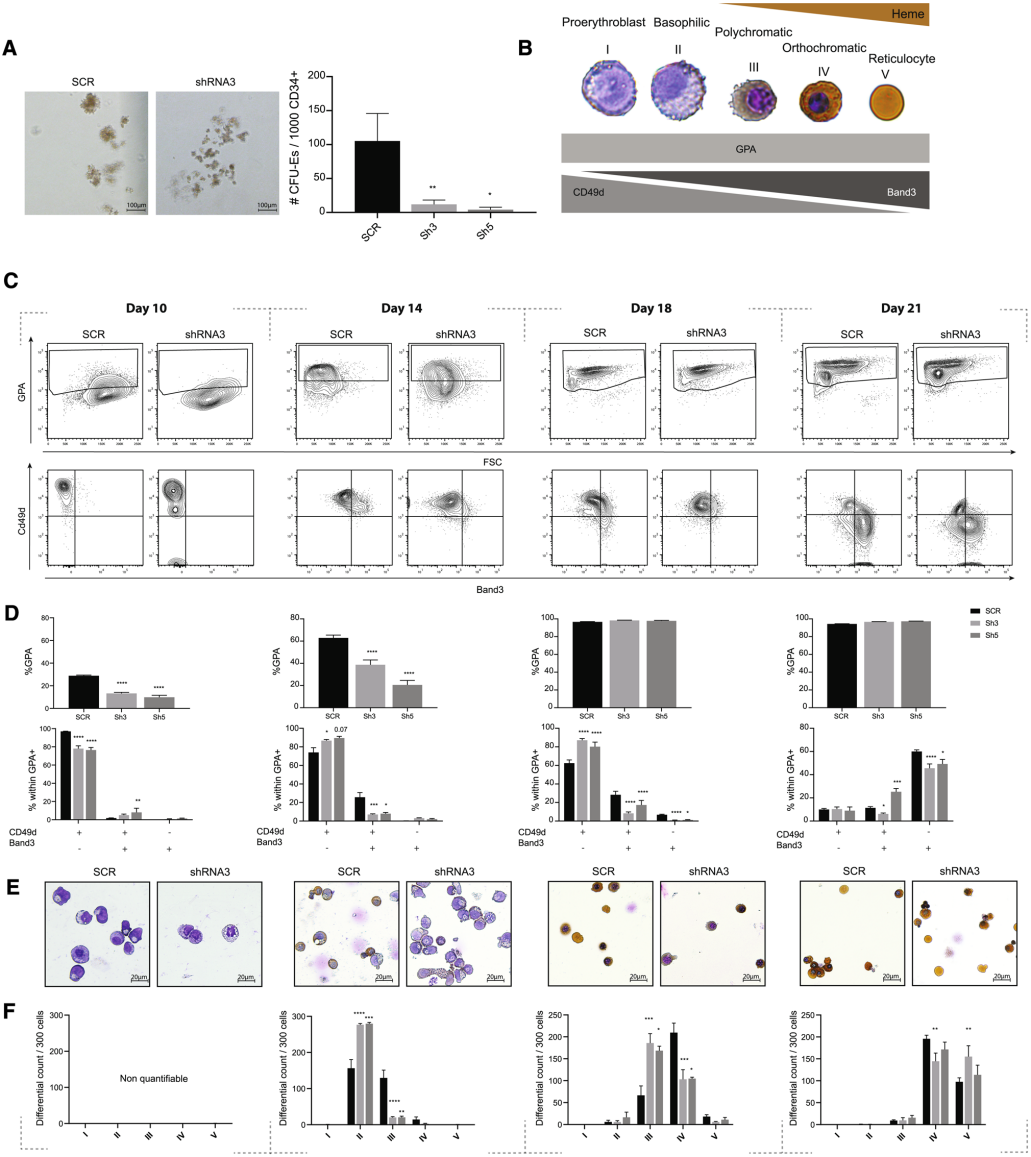
Decreased expression of PGC1β results in perturbed formation of early erythroid progenitors and delayed terminal differentiation in BM. (**A**) Representative pictures (left) and quantification (right) of CFU-E colonies at day 14 (n = SCR:4, Sh3:12, Sh5:6). (**B**) Schematic description of the cell morphology, cell surface markers and heme availability during terminal erythroid differentiation. The representative pictures are of erythroid progenitors from the erythroid culture, stained with DAB-Giemsa. (**C**) Representative plots and (**D**) quantification of flow cytometry analysis of GPA (top) and CD49d/Band3 within GPA ^+^  (bottom) on day 10, 14, 18 and 21 of erythroid in vitro differentiation (n = SCR:4, Sh3:12, Sh5:6). (**E**) Representative pictures at 40 × magnification and (**F**) quantification of progenitors from cytospins stained with DAB-Giemsa (n = 3–4). Quantification was not possible on day 10 as GPA^+^ and GPA^−^ progenitors cannot be differentiated. Data is presented as mean ± SEM (*P ≤ 0.05, **P ≤ 0.01, ***P ≤ 0.001, ****P ≤ 0.0001).

To investigate the role of PGC1β during early erythroid development, transduced CD34^+^ BM stem/progenitor cells were plated in methyl cellulose and analyzed for formation of erythroid colony forming units (CFU-Es) on day 14. Decreased expression of PGC1β severely affected the capacity of multipotent CD34^+^ progenitors to form CFU-Es, with a striking 8.8 and 25.5-fold reduced CFU-E-formation for sh3 and sh5 respectively (Fig. 2A). To further understand the importance of PGC1β during terminal erythroid differentiation, we took advantage of the pan-erythroid surface marker Glycophorin A/CD235a (GPA) in combination with differential expression of surface markers Cd49d and Band3^32^, and increased hemoglobin availability naturally occurring during stepwise erythroid maturation (Fig. 2B). Flowcytometric analysis of transduced, cultured BM cells on day 10 (GPA ^+^  cell emergence), day 14 (early erythroid differentiation), day 18 and day 21 (terminal erythroid differentiation) revealed that decreased expression of PGC1β resulted in a significant reduction in the overall formation of erythroid cells (GPA^+^) on day 10 and day 14, which was normalized by day 18 and day 21 (Fig. 2C,D).

Further fractionation of GPA ^+^  erythroid progenitors demonstrated that reduced expression of PGC1β resulted in delayed erythroid maturation, with increasing accumulation of basophilic/polychromatic erythroblasts (Cd49d^+^/Band3^−^) and impaired formation of more mature polychromatic/orthochromatic erythroblasts (Cd49d^+^/Band3^+^) on day 14 and day 18 of culture respectively (Fig. 2C,D). Erythroid differentiation was close to normalized on day 21, when the majority of cells in the culture had reached final maturation (orthochromatic erythroblasts/Reticulocytes, Cd49d^−^/Band3^+^). Terminal erythroid maturation was further analyzed morphologically, allowing more detailed fractionation. Morphological scoring demonstrated that the surface markers Cd49d and Band3 mark a continuum of differentiating erythroblasts. Furthermore, the intensity of Band3 doesn’t increase substantially between day 14 and day 18, although the cells contained in the Cd49d^+^/Band3^−^ compartment are significantly more mature on d18 (polychromatic erythroblasts) than on day 14 (basophilic). In accordance with the flowcytometric results, scoring of cells stained with Giemsa together with hemoglobin-marking 3,3′-Diaminobenzidine (DAB) (Fig. 2E) demonstrated a delay in maturation with accumulations of basophilic erythroblasts (II) on day 14, and at the polychromatic erythroblast stage (III) on day 18 (Fig. 2F). Again, the formation of more mature orthochromatic erythroblasts and reticulocytes (IV, V) was close to normalized by day 21 (Fig. 2F). The DAB-Giemsa quantification was not possible on day 10, since GPA^+^ and GPA^−^ progenitors could not be distinguished morphologically.

**Figure 3:**
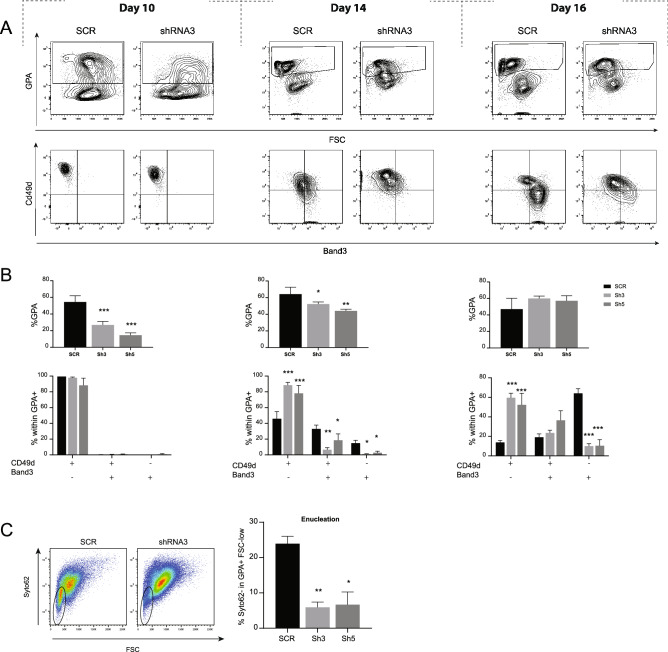
Decreased expression of PGC1β in CB derived CD34 + cells results in perturbed formation of early erythroid progenitors and impaired enucleation. (**A**) Representative plots and (**B**) quantification of flow cytometry analysis of GPA (top) and CD49d/Band3 (bottom) on day 10, 14 and 16 of CB derived CD34 + erythroid in vitro differentiation (n = 4). (**C**) Representative plots (left) and quantification (right) of enucleation efficiency based on Syto62 nucleic acid stain within mature GPA ^+^ cells (n = 4). Data is presented as mean ± SEM (*P ≤ 0.05, **P ≤ 0.01, ***P ≤ 0.001).

Differentiation from CD34^+^ cells to mature erythroid progenitors occurs faster in CB compared to BM. As a result, analyses for CB were done on day 10, 14 and 16, with day 16 corresponding to between day 18 and day 21 of BM differentiation. Knock-down of PGC1β in CB derived CD34^+^ cells resulted in a developmental delay that was even more pronounced compared to BM, with remaining significantly reduced levels of Cd49d^−^/Band3^+^ progenitors on day 16 (Fig. 3A,B). The more pronounced reduction of formed GPA ^+^   cells during early erythropoiesis using sh5 as compared to sh3 can likely be attributed to the lower PGC1β protein levels caused by sh5-mediated knock-down. Analysis of enucleation using the nucleic acid stain Syto62 demonstrated that knock-down of PGC1β also resulted in significantly reduced enucleation in mature GPA ^+^  cells compared to control (Fig. 3C). In conclusion, reduced expression of PGC1β results in perturbed formation of early erythroid progenitors and impaired terminal erythroid differentiation with an accumulation of polychromatic erythroblasts and a subsequent reduction of more mature erythroblasts, a phenotype that is reminiscent of the refractory anemia seen in many MDS patients^27^ and the MDS-like anemia we have previously reported on in mouse^21^.

### PGC1β knock-down results in deregulated hemoglobinization of erythroid progenitors

**Figure 4:**
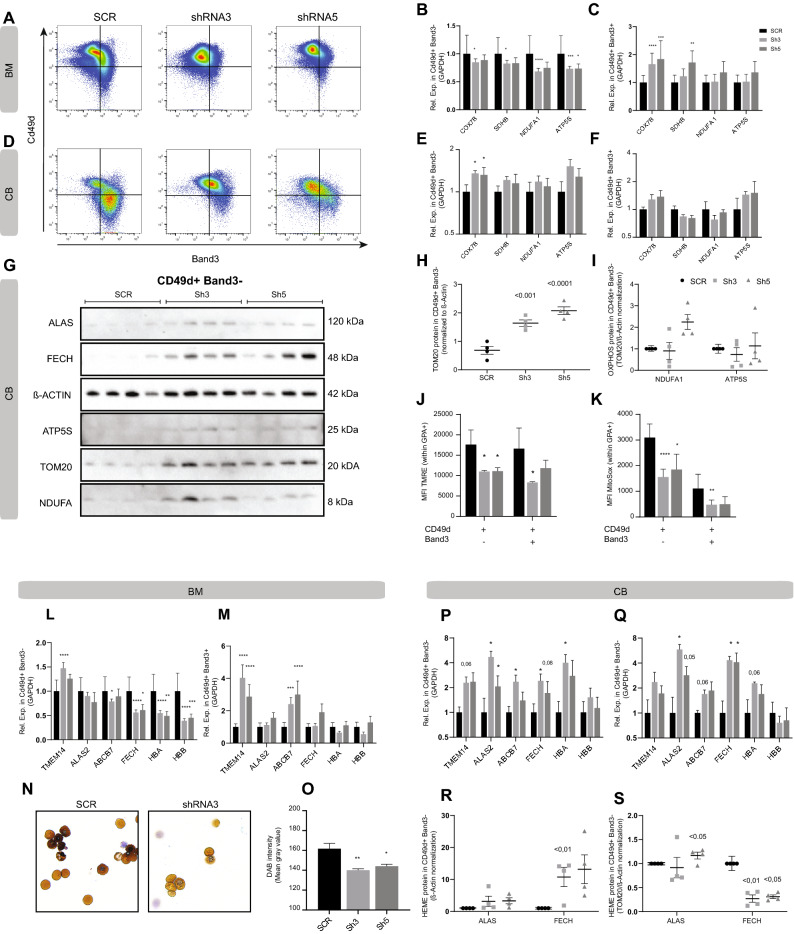
PGC1β knock-down results in deregulated hemoglobinization of erythroid progenitors. (**A**) Representative flow cytometry plots of bone marrow derived erythroid progenitors within GPA ^+^  (SCR). (**B**,** C**) RT-qPCR analysis of gene expression relative to GAPDH on day 18 of genes important for Oxidative phosphorylation (OXPHOS) in bone marrow derived (**B**) polychromatic erythroblasts and (**C**) orthochromatic erythroblasts respectively (n = SCR:4, Sh3:12, Sh5:6). (**D**) Representative flow cytometry plots of cord blood derived erythroid progenitors within GPA ^+^ (SCR). (**E**, **F**) RT-qPCR analysis of gene expression relative to GAPDH on day 16 of genes important for Oxidative phosphorylation (OXPHOS) in cord blood derived (**E**) polychromatic erythroblasts and (**F**) orthochromatic erythroblasts respectively (n = 4). (**G**) Mitochondria related protein levels were evaluated by western blot in sorted cord blood derived polychromatic erythroblasts from day 16 of culture (ALAS2 (65 kDa) specific bands appear at 120 kDa, likely due to protein dimers). Western blot quantification of (**H**) TOM20 relative to β-Actin as a marker for mitochondrial mass in relation to cell number, and (**I**) OXPHOS related ATP5S and NDUFA1 relative to TOM20/β-Actin to account for differences in mitochondrial mass. Expression depicted as normalized to control (SCR, n = 4). (**J**) Quantification of mitochondrial membrane potential of cord blood derived polychromatic erythroblasts measured on day 16 of differentiation using TMRE and flow cytometry (n = 4). (**K**) Quantification of ROS production by mitochondria in cord blood derived polychromatic erythroblasts measured on day 16 of differentiation using MitoSox and flow cytometry (n = 4). (**L**, **M**) RT-qPCR analysis of gene expression relative to GAPDH on day 18 of genes important for hemoglobin and heme synthesis of bone marrow derived polychromatic erythroblasts (left) and orthochromatic erythroblasts (right) respectively (n = SCR:4, Sh3:12, Sh5:6). (**N**) Representative images of DAB-Giemsa stained erythroblasts from d21 of culture at 40 × magnification and (**O**) quantification of hemoglobin content using ImageJ software (n = SCR:4, Sh3:12, Sh5:6). (**P**, **Q**) RT-qPCR analysis of gene expression relative to GAPDH on day 16 of genes important for hemoglobin and heme synthesis in cord blood derived polychromatic erythroblasts and orthochromatic erythroblasts respectively (n = 4). (**R**) Western blot quantification of heme related ALAS2 and FECH relative to β-Actin and (**S**) TOM20/β-Actin to account for differences in mitochondrial mass (n = 4). Expression depicted as normalized to control (SCR. Data is presented as mean ± SEM (*P ≤ 0.05, **P ≤ 0.01, ***P ≤ 0.001, ****P ≤ 0.0001).

The functional role of the mitochondrial coactivator PGC1β in erythropoiesis remains largely unknown. However, we recently showed that down-regulation of *pgcl*β in response to *pRb* deletion correlated with decreased expression of genes involved in mitochondrial function, as well as heme synthesis and iron transport in mouse^21^, and compound deletion of the related *Pgc1*α and *Pgc1*β genes in mouse has indicated that PGC1 coactivators might be able to affect globin gene transcription during fetal development^26^. Furthermore, mitochondrial clearance has been shown to be important for human terminal erythroid differentiation, with mitochondrial retention due to deregulated mitophagy resulting in perturbed enucleation^14,15^. To investigate mitochondrial effects of reduced PGC1β expression during human erythroid differentiation, we analyzed the expression of key genes involved in oxidative phosphorylation (OXPHOS) in BM derived erythroid progenitors on day 18 (Fig. 4A–C) and CB derived erythroid progenitors on day 16 (Fig. 4D–F). In BM, decreased PGC1β expression resulted in decreased expression of *COX7B* (Cytochrome C Oxidase, respiratory electron transport^34^), *SDHB* (CII: respiratory electron transport^35^), *NDUFA1* (CI: respiratory electron transport^36^) and *ATP5S* (ATP synthesis^37^) in polychromatic erythroblasts (Fig. 4B). In contrast, the expression of *COX7B* and *SDHB* were increased while *NDUFA1* and *ATP5S* were unchanged in the more mature orthochromatic erythroblasts (Fig. 4C). In CB derived progenitors (Fig. 4D), displaying a similar developmental delay, the transcriptional profile of OXPHOS genes in polychromatic erythroblasts more resembled that of later maturation stages in the BM (Fig. 4C), with increased expression of only *COX7B* (Fig. 4E), while none of the investigated genes were differentially expressed in the orthochromatic erythroblasts (Fig. 4F). This fits well with that CB derived progenitors have differentiated further on day 16 than BM progenitors on d18 (Fig. 4A,D).

As many OXPHOS genes are tightly regulated at the post translational level western blot analysis on key mitochondria related proteins was performed on CB-derived sorted erythroid progenitor populations from day 16 (Fig. 4G and S4A, full blots in Figs. S2, S3). To investigate the levels of mitochondrial biomass compared to cell mass, the protein level of the mitochondrial marker TOM20 was analyzed in relation to β-Actin. In accordance with what we have previously shown in mouse^21^ and Moras et al., have demonstrated in human cells^14^, mitochondrial biomass decreased during the final maturation steps of unperturbed terminal erythroid differentiation, to become virtually non-detectable at the orthochromatic stage (Scr in Figs. 4G,H and S4A), indicating that a significant portion of the mitochondria have been cleared at that stage. Interestingly, TOM20 was detected at significantly increased levels in the sh PGC1β transduced polychromatic erythroblasts (Fig. 4H). A difference that was no longer apparent in the orthochromatic erythroblasts (Fig. S4A), suggesting a delay in mitochondrial clearance. This delay was also apparent in BM derived erythroid progenitors, where knock-down of PGC1β resulted in increased mitochondrial biomass in all progenitor populations during early differentiation (day 14) as analyzed by flow cytometry and Mitotracker Deep Red (MTDR) (Fig. S4B), which was less pronounced on day 18 (Fig. S4B).

Further analysis of OXPHOS related proteins revealed that NDUFA1 and ATP5S were not significantly altered in sh PGC1β transduced cells, when taking into account the increased mitochondrial biomass (Fig. 4I). However, if instead comparing to cell numbers (β-ACTIN), sh3 transduced cells had significantly increased NDUFA1 protein (Fig. S4C). To evaluate mitochondrial function, CB derived erythroid progenitors were stained with TMRE and MitoSox on day 14 and 16 of differentiation, to measure mitochondrial membrane potential and mitochondrially produced reactive oxygen species (ROS) respectively. While decreased expression of PGC1β resulted in increased mitochondrial membrane potential on day 14, especially using sh3, (Fig. S4D), the mitochondrial membrane potential was significantly reduced at day 16 in both sh PGC1β transduced polychromatic erythroblasts, and in the sh3 transduced orthochromatic erythroblasts (Fig. 4J), in spite of having more mitochondrial mass per cell (Fig. 4h). At day 16 of differentiation, the mitochondrial membrane potential nicely correlated with the level of mitochondrial ROS production (Fig. 4K). This was also true for the control cells on day 14 (Fig. S4D,E), while the levels of mitochondrial ROS increased significantly with differentiation in cells transduced with sh3, despite decreasing membrane potential (Fig. S4E). In conclusion, reduced levels of PGC1β results in disruption of mitochondrial clearance. Furthermore, PGC1β knock-down leads to reduced expression of key OXPHOS genes in earlier erythroid progenitors, but increased expression in more mature progenitors. Notably, mitochondrial function is reduced in both polychromatic and orthochromatic erythroblasts despite increased mitochondrial biomass.

We next sought to understand the involvement of PGC1β in regulation of hemoglobin composition and iron transport, biological processes taking place in the mitochondria that are fundamental for proper red blood cell function. Transcriptional analysis of BM derived erythroid progenitors on day 18 revealed that the expression of *TMEM14C* (transporter of protoporphyrinogen^38^) was increased, *ALAS2* (the rate-limiting enzyme in heme synthesis^39^) remained unchanged, while the levels *ABCB7* and *FECH* (catalyzing insertion of iron into heme^40^), were decreased in response to PGC1β knock-down in polychromatic erythroblasts (Fig. 4L). Furthermore, in accordance with Cui et al.^26^, gene expression of both globin *HBA* and *HBB* subunits was significantly reduced (Fig. 4L). In the more mature orthochromatic erythroblasts, the expression of *ALAS2*, *FECH*, *HBA* and *HBB* was unchanged, while *TMEM14C* and *ABCB7* had increased expression compared to control (Fig. 4M). To functionally investigate if reduced expression of genes regulating heme synthesis and iron transport had an effect on hemoglobin content, erythroid cells from day 21 of culture were stained with hemoglobin-marking DAB (Fig. 4N). Analysis of DAB-intensity as a proxy for heme content revealed a 13% and 11% decrease in the mature reticulocytes with sh3 and sh5 knock-down respectively compared to control (Fig. 4O). Transcriptional analysis in CB derived polychromatic erythroblast on day 16 instead revealed an increased expression of several of the heme related genes, which was especially prominent in sh3 transduced cells (only a significant for *ALAS2* using sh5) (Fig. 4P), potentially reflecting that CB derived progenitors have differentiated further on day 16 than BM progenitors on d18 (Fig. 4A,D). In the orthochromatic erythroblasts only *ALAS2* and *FECH* remained significantly increased (Fig. 4Q). ALAS2 and FECH were further analyzed using western blot (Figs. 4G, S4A). When compared to total cell numbers (β-ACTIN) FECH protein was significantly increased in sh3 transduced polychromatic erythroblasts (Fig. 4R), in agreement with the transcriptional data. However, when taken the increased mitochondrial biomass into account, FECH protein was instead significantly reduced in sh PGC1β transduced cells, while ALAS2 remained slightly increased in sh5 transduced polychromatic erythroblasts (Fig. 4S). In agreement with clearance of mitochondria in later erythroid maturation stages (no detectable TOM20), only FECH had clear bands in orthochromatic erythroblasts (Fig. S4A) with a trend towards increased protein compared to control (Fig. S4F). In conclusion, reduced levels of PGC1β results in deregulation of genes involved in hemoglobin composition and iron transport, as well as reduced hemoglobin content during the final stages of erythroid maturation in BM.

### PGC1β knock-down results in disturbed cell cycle exit and smaller reticulocytes

**Figure 5:**
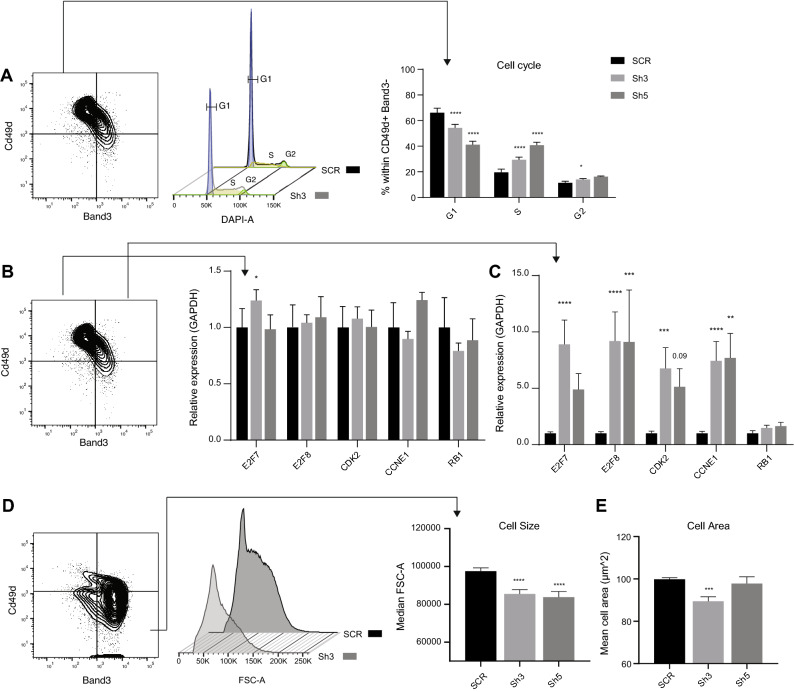
PGC1β knock-down results in perturbed cell cycle exit and smaller reticulocytes. (**A**) Representative plots indicating populations analyzed (SCR, left) and cell cycle profile (middle), and quantification (right) of the cell cycle status on d18 within bone marrow derived polychromatic erythroblast (CD49d^+^ Band3^−^), using flow cytometry and DAPI (n = SCR:4, Sh3:12, Sh5:6). (**B**, **C**) Analysis of expression relative to GAPDH on day 18 of cell cycle related genes involved in G1 to S phase progression in (**B**) polychromatic erythroblasts (CD49d^+^ Band^−^), and (**C**) orthochromatic erythroblasts (CD49d^+^ Band^+^) using RT-qPCR (n = SCR:4 , Sh3:12, Sh5:6). (**D**) Representative plot indicating populations analyzed (SCR, left) and quantification (right) of cell size using flow cytometry and FSC-A within orthochromatic erythroblasts (CD49d^-^ Band^+^) on day 21 at the final stage in terminal differentiation. (**E**) 2D cell area quantification of enucleated cells (day 21) stained with DAB-Giemsa, using cytospin microscopy images and ImageJ software, (n = SCR:4, Sh3:12, Sh5:6). Data is presented as mean ± SEM (*P ≤ 0.05, **P ≤ 0.01, ***P ≤ 0.001, ****P ≤ 0.0001).

Activation of PPAR signaling has been found to be involved in induction of cell cycle arrest in adipocytes^41^ and inhibition of cell growth in human colorectal cancer cell lines^42^. In addition, we recently demonstrated in mouse that down-regulation of PGC1β in response to *pRb* deletion correlated with enhanced cycling and increased expression of cell cycle genes in erythroid progenitors^21^. Importantly, overexpression of *Pgc1β* could normalize the deregulated expression of cell cycle genes induced by pRb deficiency^21^. To further study the direct role of PGC1β in cell cycle regulation in human erythropoiesis, polychromatic erythroblasts from day 18 of culture were stained with DAPI and analyzed for cell cycle status using FACS (Fig. 5A). Since erythroid cells are sensitive to permeabilization and fixation, the polychromatic erythroblasts were sorted prior to fixation and staining. In accordance with our previous results in mouse^21^, decreased expression of PGC1β resulted in fewer polychromatic erythroblasts in G1-phase and a 1.5- and twofold increase in cells remaining in S-phase for sh3 and sh5 respectively compared to control (Fig. 5A). Transcriptional analysis on day 18 of genes involved in G1 to S progression revealed that, while the expression of these genes was largely unchanged polychromatic erythroblasts (Fig. 5B), the expression of *E2F7* and *E2F8* (repression of G1/S-regulated genes^43^), *CDK2* (G1/S transition^44^), and *CCNE1* (regulator of *CDK2*^45^) were greatly upregulated in the slightly more mature orthochromatic erythroblasts (Fig. 5C). The same cell cycle related genes were also up-regulated in CB derived erythroblasts on day 16 already at the polychromatic erythroblasts stage (Fig. S5), demonstrating that reduced levels of PGC1β affects cell cycle regulation in both BM and CB erythropoiesis, in spite of known differences in cell cycle status between these hematopoietic tissues^46^.

**Figure 6:**
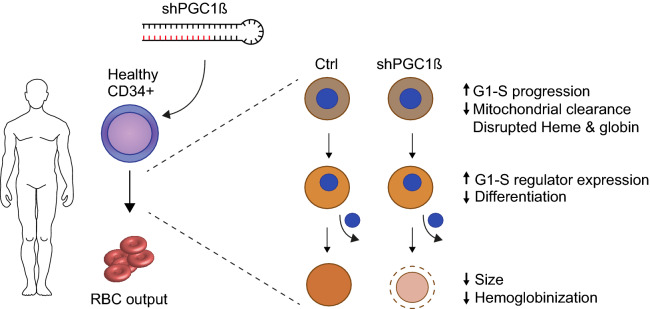
PGC1β is directly involved in mitochondrial clearance, production of hemoglobin and regulation of G1-S transition and is ultimately required for proper terminal erythroid differentiation.

It has been shown that erythroid precursors that undergo fewer cell divisions result in larger erythrocytes^47^. To investigate if enhanced cycling resulted in reduced cell size, we quantified the size of the orthochromatic erythroblasts/Reticulocytes (CD49d^−^ Band3^+^) on day 21 using Forward Scatter Area (FSC-A) and flow cytometry. Results clearly demonstrated that reduced expression of PGC1β resulted in a 13.6% (sh3) and 8.4% (sh5) decrease in median FSC-A compared to cells transduced with control vector (Fig. 5D). Similarly, quantification of the 2D area of reticulocytes from cytospin images at day 21 of culture using ImageJ^48,49^ demonstrated a 10% decrease in cell area using the more efficient sh3, while there was no significant difference in reticulocyte size observed using sh5 (Fig. 5E). Taken together, our results demonstrate that decreased expression of PGC1β results in perturbed human erythroid differentiation, reduced mitochondrial clearance, disrupted hemoglobin production, inability to properly exit cell cycle, and a consequent reduced cell size of reticulocytes (Fig. 6).

## Discussion

Multiple pathways involved in cell cycle regulation, organelle clearance, apoptosis and iron homeostasis are crucial for functional erythropoiesis^50–53^. A central player for several of these pathways is the mitochondria. However, mechanisms regulating mitochondrial function during erythropoiesis remains elusive. Here we demonstrate that decreased expression of PGC1β a critical coactivator of mitochondrial biogenesis, results in perturbed formation of early erythroid progenitors and delayed human erythroid differentiation, reduced reticulocyte size, disturbed cell cycle regulation and deregulated hemoglobinization. Knock-down of PGC1β expression resulted in a substantial and similar phenotype with both short hairpins used, in spite of their different and relatively low knock-down efficiencies (sh3: 50%, sh5: 20%), demonstrating the importance of unperturbed levels of PGC1β for both early and terminal erythroid development. shRNA mediated gene silencing can either degrade mRNA or inhibit protein translation. The weaker effect on mRNA transcription, but stronger reduction at the protein level detected using sh5, could hence potentially be due to sh5 exerting its effect mainly through stronger inhibition of protein translation.

PGC1α has been shown to have a compensatory effect in other tissues in Pgc1β KO mice^54^. We therefore investigated if reduced expression of PGC1β resulted in a compensatory increase in PGC1α in the human setting. PGC1α expression, which was undetectable in wildtype ortho/poly chromatic erythroblasts, was however not upregulated in response to reduced levels of PGC1β (data not shown), ruling out compensatory mechanisms between the two homologs in the erythroid compartment.

Being a coactivator of mitochondrial biogenesis, it could be expected that reduced expression of PGC1β would result in decreased expression of OXPHOS related genes and proteins. In contrast, mitochondrial biomass was increased in all BM derived erythroblast populations on day 14 as measured by MTDR. This is likely due to disrupted mitophagy. During terminal erythroid differentiation mitochondria undergo mitophagy leading to decreased MTDR intensity^14,55^, which is also evident by our data, demonstrating a stepwise reduction of MFI in maturing erythroblasts in the control cells (Fig. S4B, SCR black bars). This is also in agreement with our data showing that the mitochondrial housekeeping protein TOM20 is present in polychromatic erythroblasts (Fig. 4G,H), but no longer detectable in orthochromatic erythroblasts (Fig. S4A). That PGC1β likely plays a functional role in mitochondrial clearance was further strengthened by that knock-down of PGC1β resulted in retention of TOM20 expression in CB derived polychromatic progenitors compared to control (Fig. 4G,H). However, the fact that the same cell populations display reduced TMRE and MitoSox intensity in spite of increased mitochondrial cell mass, indicate that the retained mitochondria are not fully functional. We further demonstrate that knock-down of PGC1β results in significantly reduced enucleation in mature GPA^+^ cells compared to control. Interestingly, it was recently shown that late-stage erythroblasts sustain mitochondrial metabolism and subsequent enucleation, and that metabolic fueling of mitochondria is critical for the erythroid enucleation process^56^.

The perturbed formation of erythroid cells from BM was the most prominent during early differentiation, whereas the formation of mature reticulocytes was close to normalized by day 21 of culture. The recovery noted on day 21 indicate that PGC1β might be less important during the final maturation steps of adult erythropoiesis. It could however also be due to cell intrinsic attempts to compensate. For example, the decreased expression of heme-, globin- and some OXPHOS-related genes seen in response to PGC1β knock-down in polychromatic erythroblasts, was normalized or even increased in the more mature orthochromatic erythroblasts. Erythroid development from CB progenitors was even more affected by the decreased levels of PGC1β indicating that PGC1β plays a more prominent role during earlier ontogeny. In contrast to what was seen in adult erythroblasts, CB derived erythroblasts, displayed increase expression of heme- and globin related-genes. This could potentially be due to the retention of mitochondria, or a cell intrinsic attempt to compensate for deregulated heme-regulation at earlier stages of differentiation, as seen in BM.

Erythropoiesis is intimately coupled to cell cycle regulation, and erythroblasts undergo initial self-renewal divisions, followed by a series of quick divisions during terminal differentiation to give rise to the immense amounts of mature RBCs each day^57,58^. In addition to its more established role as regulator of mitochondrial biogenesis and energy metabolism, PGC1β has been implicated in cell cycle regulation of cancer cells^42^. Furthermore, we recently showed that enhanced Pgc1β expression alone was enough to normalize the disrupted expression of cell cycle related genes in pRb-deficient murine erythroblasts^21^. Here we show that multiple genes involved in G1 to S progression were greatly increased in response to PGC1β knock-down in both BM and CB derived erythroblasts, demonstrating a direct involvement of PGC1β in cell cycle regulation. While the total expression of *pRB*, a G1-S master regulator^59^, was unchanged, *CDK2* that targets pRB for phosphorylation^60^ was significantly increased. Hence, hyper-phosphorylation of pRB by CDK2 could potentially contribute to increased cell cycle progression in PGC1β-deficient cells. Furthermore, in line with a previous publication showing that erythroid precursors that undergo fewer cell divisions result in larger erythrocytes^47^, we here demonstrate that increased cell cycle progression results in smaller reticulocytes.

The disrupted terminal erythroid differentiation with accumulations of polychromatic and orthochromatic erythroblasts that we see in response to decreased levels of PGC1β is reminiscent of the impaired terminal erythroid differentiation seen in MDS^27^. The majority of MDS patients are transfusion dependent, which negatively affect their quality of life, clinical outcome and correlates with increased risk of leukemic transformations^61–64^. A high frequency of acquired mutations in mitochondrial DNA has been reported in MDS patients^29^. For example, subtypes of MDS with somatic SF3B1 mutations are associated with downregulation of core mitochondrial pathways^30,31^. This includes deregulation of genes important for iron and heme homeostasis^31,65^, suggesting that these biological processes could contribute to the disease phenotype. Notably, PGC1β is located within the commonly deleted region of chromosome 5 of the del(5q) subtype of MDS^66^. While the pathogenesis of del(5q) MDS has been attributed to heterozygous deletion of RPS14^67^, it is unlikely that the full disease phenotype is caused by the loss of a single gene out of the 40 coding genes included in the commonly deleted region^66^. Patients with del(5q) MDS exhibit a similar inefficient erythropoiesis^68^ to that described here, indicating that PGC1β deficiency potentially also contributes to the disease phenotype. In addition, reduced PGC1β expression result in disrupted heme and globin coordination, which is also reported in del(5q) MDS^68^. Furthermore, Lenalidomide, which is used to treat del(5q) MDS patients^69^, has been shown to also affect mitochondrial homeostasis^70^. However, further studies are needed to determine if disturbed PPAR-signaling is involved in MDS-related anemia pathogenesis.

## Materials and methods

### Human BM cells

Human bone marrow (BM) cells were supplied by Lonza or collected at the Hematology Department, University of Lund, Sweden. Informed consent was obtained from all participants. All work on BM samples was done according to ethical guidelines approved by the Institutional Review Board of the University of Lund and in accordance with the Declaration of Helsinki.

### Vector design and transduction

Bacterial glycerol stocks with shPGC1β inserts from MISSION® sh3: TRCN0000008600 and sh5: TRCN0000429958 clones (Sigma Aldrich, St. Louis, MO, USA) or scrambled for control, as described in Supplemental Table 1, were cloned into pLKO lentiviral vector with eGFP reporter gene using NdeI and BamHI restriction enzymes. Control digestions on plasmid were done with NdeI and BamHI prior to sequencing. Cloned plasmids were sent with U6 FWD primer to Eurofins Scientific (Luxembourg) for plasmid sequence confirmation.

Approximately 100,000 CD34^+^ cells were transduced with shPGC1β lentiviral vectors or scramble control vector in phase I medium (see below) supplemented with 0.4 µg/mL Protamine sulphate. Upon validation of different shPGC1β vectors, variance was observed in knock-down efficiency between transduction replicates of the same vector and donor. To ensure sufficient knock-down efficiency for all donor samples, more transductions were performed with the shPGC1β vectors as compared to the control Scramble vector. This was done to avoid losing biological replicates to inefficient transductions Transduced (GFP^+^) CD34^+^ (APC-eFluor780, Thermo Fisher Scientific, Waltham, MA, USA) cells were sorted into RLT buffer (1% β-mercaptoethanol) 48 h after transduction for analysis of PGC1β knockdown efficiency.

### Erythroid culture

Transduced CD34^+^ bone marrow cells (Lonza, Basel, Switzerland) were sorted 48 h post transduction on Aria III based on GFP and CD34 (4H-11, APC-eFluor780, Thermo Fisher Scientific) expression and cultured in a 3-phase erythroid culturing system; 5 days in phase 1 medium, consisting of SFEM supplemented with 1% penicillin streptomycin , 50 ng/ml SCF, 10 ng/ml IL-3, 50 ng/ml FLT3, 20 ng/ml TPO and 100 nM Dexamethasone, followed by 7 days in phase 2 medium (SFEM with 1% penicillin streptomycin, 50 ng/ml SCF, 10 ng/ml IL-3, 50 ng/ml, 2U/ml EPO and 100 nM Dexamethasone) for differentiation of erythroid committed progenitors. On Day 14 the medium was switched to phase 3 medium (SFEM with 1% penicillin streptomycin, 30% heat inactivated FBS and 3U/ml EPO) for terminal erythroid differentiation. Cells were collected on day 10, 14, 18 and 21 (BM) and day 10, 14 and 16 (CB) for morphologic and flow cytometric analysis. Cells collected on day 18 for BM and day 16 for CB were also sorted for RT-qPCR, Western analysis (CB) and cell cycle analysis (BM).

### Erythroid colony formation assay

1000 transduced CD34^+^ cells were plated in MethoCult™ (H4230, Stemcell Technologies) supplemented with 20 ng/ml SCF, 10 ng/ml IL3, 50 ng/ml GM-CSF, 3U/ml EPO, incubated at 37 °C and 5% CO_2_ and scored for CFU-E colonies after 14 days.

### Morphologic scoring of diaminobenzidine (DAB) stained BM derived erythroid progenitors

Approximately 10,000–15,000 cells were spun down on cytospins slides and fixed with methanol at − 20 °C for 2 min. The slides were air-dried and stained with Diaminobenzidine hydrochloride (DAB) solution (10 mg/ml in PBS, Sigma Aldrich) including freshly added H_2_O_2_ (3 ppm, Sigma Aldrich). DAB solution was applied on the slides with the help of a PAP pen to cover all cells. The slides were washed with DH2O, stained with Giemsa (1:20 dilution in distilled water) for 15 min, wash with DH2O, and air-dried. 300 cells per transduction were visually scored into five different developmental stages based on known morphologic characteristics including presence and size of nucleus, hemoglobin content and general cell size, using an Olympus BX43 microscope (Olympus Corporation, Tokyo, Japan) and 40 × magnification. Apoptotic cell displaying blebbing and or fragmentation were excluded.

Cell area and hemoglobin stain intensity was scored using ImageJ^48,49^ (v1.153c). For quantification of cell size, images were converted to grayscale by changing type to 8-bit and converted to binary format. Cell area was quantified using ‘Analyze particles’ with a threshold of 50–150 µm^2^. For quantification of hemoglobin content, reticulocytes were marked with selection tool, followed by subtraction of noise, grayscale conversion and inversion of image. Reticulocytes were then analyzed for DAB color intensity by measuring integrated density. For more detail see Supplemental Methods.

### Flow cytometric analysis

Cultured transduced cells were collected on days 10, 14, 18, 21 (BM) and days 10, 14, 16 (CB) for FACS analysis. The cells were washed with PBS (GE Healthcare Life Sciences) with 2% FCS (GE Healthcare Life Sciences) and stained with the cell surface markers GPA (GA-R2, BV421, BD), CD49d (9F10, PeCy7, BioLegend) and Band3 (Bric 6, PE/APC, IBGRL Research Products). Mitotracker Deep Red (50 nM, Thermo Fisher Scientific), TMRE (100 nM, Thermo Fisher Scientific) and MitoSox (250 nM, Thermo Fisher Scientific) was included to measure mitochondrial biomass, membrane potential and ROS production respectively. Nucleic Acid Stain Syto62 (25 nM) was included to assess enucleation. 7-Aminoactinomycin D (7-AAD, Sigma Aldrich) was used to exclude dead cells. Samples were analyzed and sorted on FACS Aria III, FACS Aria II and LSR Fortessa.

### Cell cycle analysis

Since erythroid cells are sensitive to permeabilization and fixation, 200,000–400,000 polychromatic erythroblasts were sorted based on their GPA, CD49d and Band3 expression prior to fixation. Cell pellets were fixed with ice cold 70% Ethanol in PBS (GE Healthcare Life Sciences) while vortexing and incubated at − 20 °C overnight. Fixed cells were washed with PBS and stained with DAPI staining buffer (0.1% TritonX100 (Sigma Aldrich), 0.1% Sodium citrate (Sigma Aldrich), 10 µg/ml DAPI (Sigma Aldrich) for 30 min at 37 °C. DAPI intensity was analyzed individually in the sorted erythroid progenitor populations on LSR Fortessa (Becton Dickinson).

### Gene expression

5000–20,000 polychromatic (Cd49d^+^, Band3^−^) and orthochromatic (Cd49d^+^, Band3^+^) erythroid progenitors were sorted into RLT buffer (1% β-mercaptoethanol) and snap frozen. RNA was purified using RNeasy mini kit according to instructions (Qiagen) and cDNA was prepared using SuperScript IV reverse transcriptase (Thermo Fisher Scientific). RT-qPCR assays were performed according to the TaqMan Gene expression master mix protocol and pre-designed probe-based primers (Supplemental Table 2) (Integrated DNA Technologies, Coralville, IA USA and Thermo Fisher Scientific).

### SDS-PAGE and immunoblotting

Whole cell lysates of transduced cord blood derived cells on day7 were used for analysis of knock-down efficiency, and GPA^+^, CD49d^+^/Band3^−^ and CD49d^+^/Band3^+^ populations were sorted on day 16 for analysis of OXHPOS and cell cycle proteins. Sorted cells were prepared with RIPA lysis and extraction buffer (Fischer scientific). Protein concentration of lysates were quantified with Pierce™ BCA Protein Assay Kit (Thermo Fisher Scientific) and proteins were separated using NuPAGE® 4–12% Bis–Tris gels. Proteins were transferred to nitrocellulose membrane using Trans-Blot Turbo Transfer System (BioRad, Hercules, CA, USA). Immunoblotting was done with following antibodies and dilutions overnight at 4 °C: Rabbit Anti-PGC1 beta Ab (ab176328, 1:1000, Abcam, Cambridge UK), Rabbit Anti-NDUFA1 Ab (ab176563, 1:1000, Abcam), Mouse Anti-TOM20 Ab (sc-17764, 1:1000, Santa Cruz Biotechnology, Dallas, TX, USA) ,Mouse Anti-ALAS-E Ab (sc-166739, 1:1000, Santa Cruz Biotechnology), Mouse Anti-Ferrochelatase Ab (sc-377377, 1:1000, Santa Cruz Biotechnology) Rabbit Anti-ATP5S Ab (PA5-101443, 1:1000, Fisher Scientific), Mouse Anti-Actin (Ab-5, 1:1000, Biosciences), HRP coupled anti-rabbit IgG (NA9340-1ML, 1:5000, Cytiva), HRP coupled anti-mouse IgG (NA9310-1ML, 1:5000, Cytiva). Proteins of interest were exposed using Amersham ECL Prime Western Blotting Detection Reagent (Cytiva, Marlborough, MA, USA). Images were acquired by ChemiDoc™ Imaging Systems (Bio-Rad) and bands intensity were quantified with Image Lab software (v6.0, Bio-Rad).

### Statistics

Statistical analysis was done using GraphPad Prism 8.00 or 9.00 (GraphPad Software, San Diego, CA USA). Significance was analyzed with 2way ANOVA test, corrected for multiple comparisons, using Sidak’s or two-stage step-up method of Benjamini, Krieger and Yekutieli (False discovery rate), if not stated otherwise. Students paired t-test was used for analyzing RT-qPCR, Enucleation, DAB intensity and Cell size analyses, when only comparing two groups.

## Supplementary Information

Supplementary Information.
