# Supplementary material for: Decreased PGC1β expression results in disrupted human erythroid differentiation, impaired hemoglobinization and cell cycle exit
Source: Sci Rep. 2021 Aug 24;11:17129. doi: 10.1038/s41598-021-96585-0 (PMC8385110; doi:10.1038/s41598-021-96585-0)

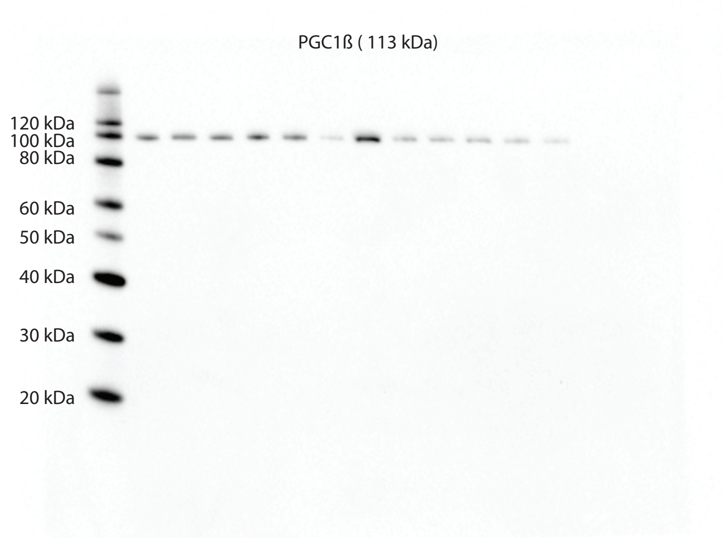

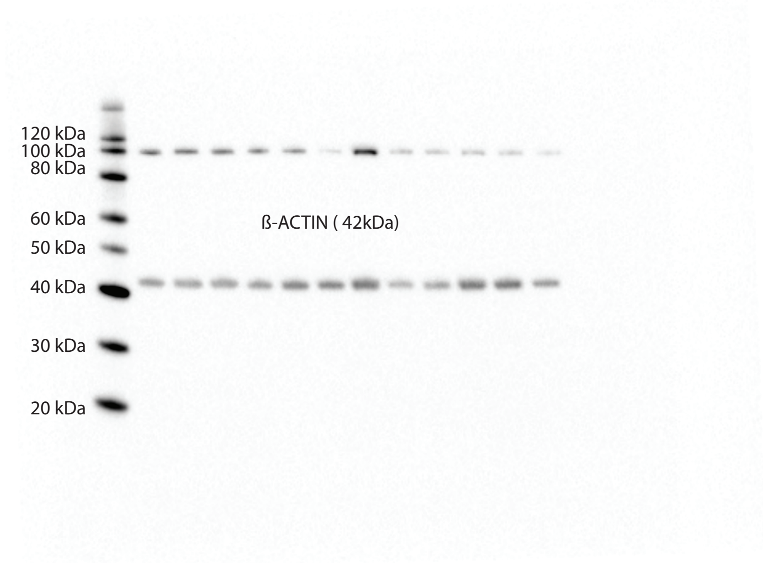

Figure S1. Full size blot images used in Figure 1 to determine PGC1ß knock-down efficiency. Anti-PGC1ß immunoblotting (left) was done prior to immunoblotting with Anti-ß-ACTIN (right).

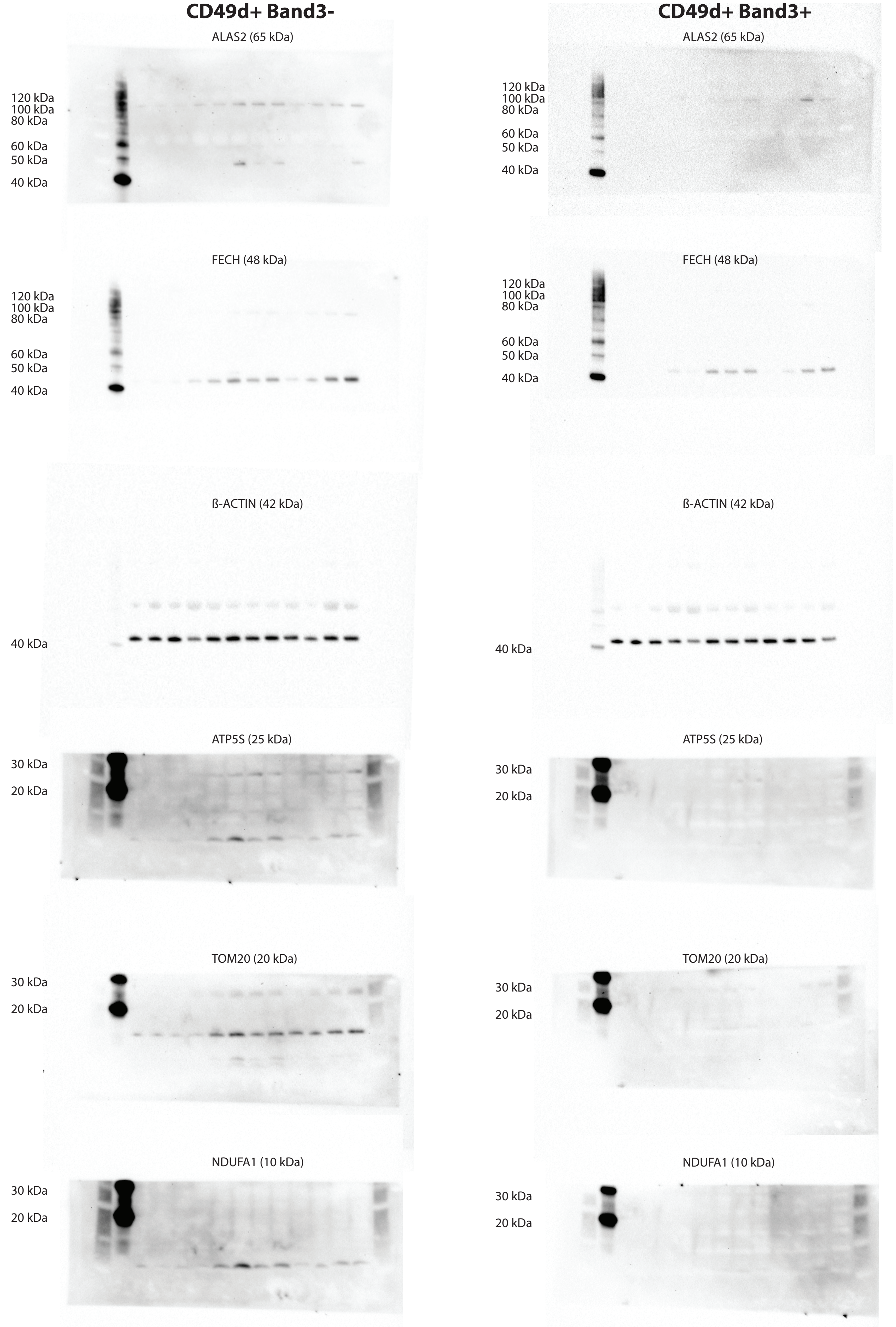

Figure S2. Full size blot images of the OXPHOS and heme realted proteins used in Figure 4 and Figure S1. CD49d+ Band3- (polychromatic erythroblasts, left) and CD49d+ Band3+(orthochromatic erythroblats, right) sorted at day 16 of differentiation. Blots were cut at 35kDa and stained with different antibodies. ALAS2 (65kDa) specific bands appear at 120 kDa, likely due to protein dimers.

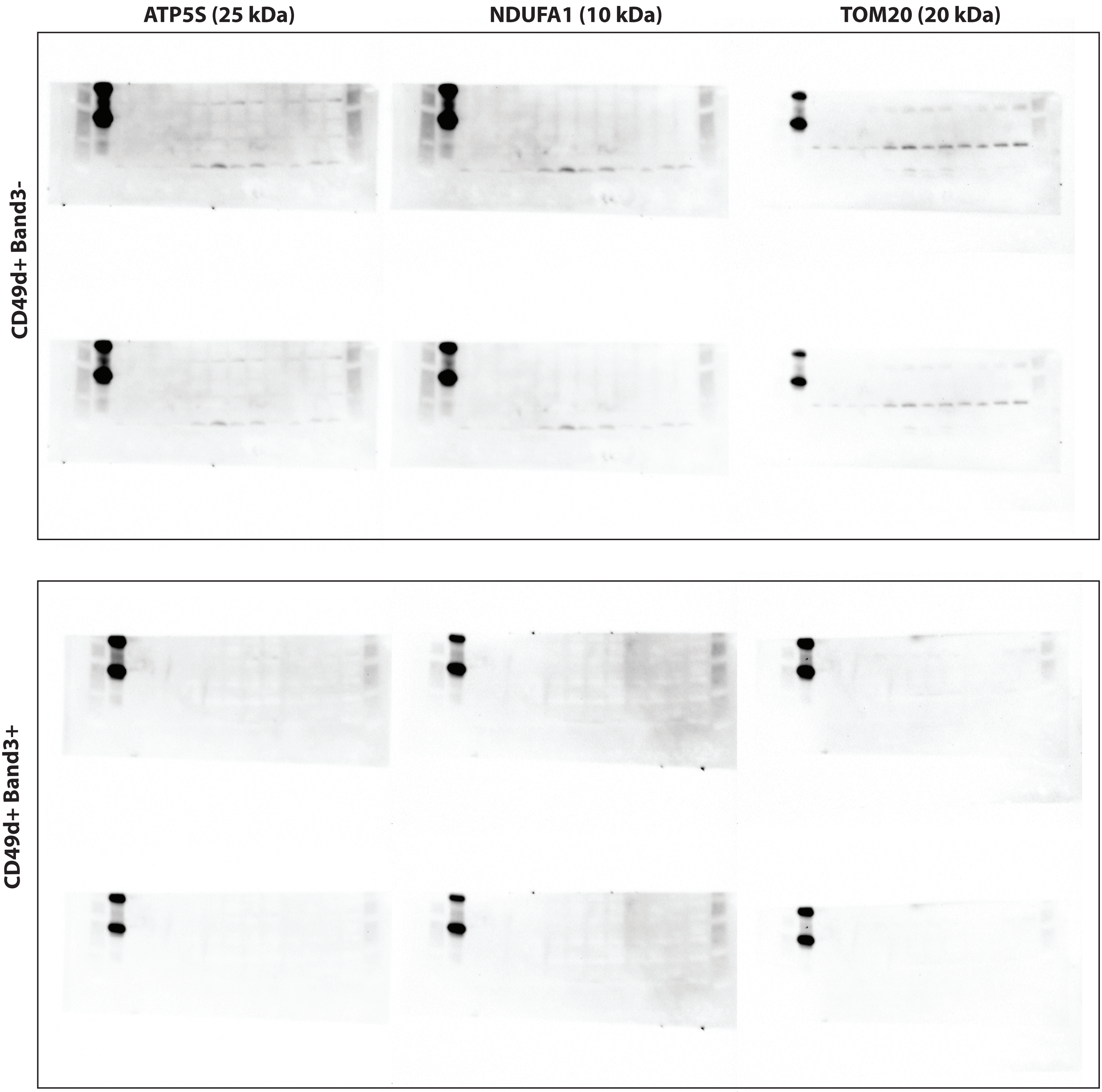

Figure S3. Full size blot images of the OXPHOS realted proteins with 2 additional lower exposed images (compared to Figure 4 and Figure S1). Due to higher contrast on ATP5S, TOM20 and NDUFA blots in figure 4G we include here same blots with lower exposure to compliment the high contrast images in Figure 4G.

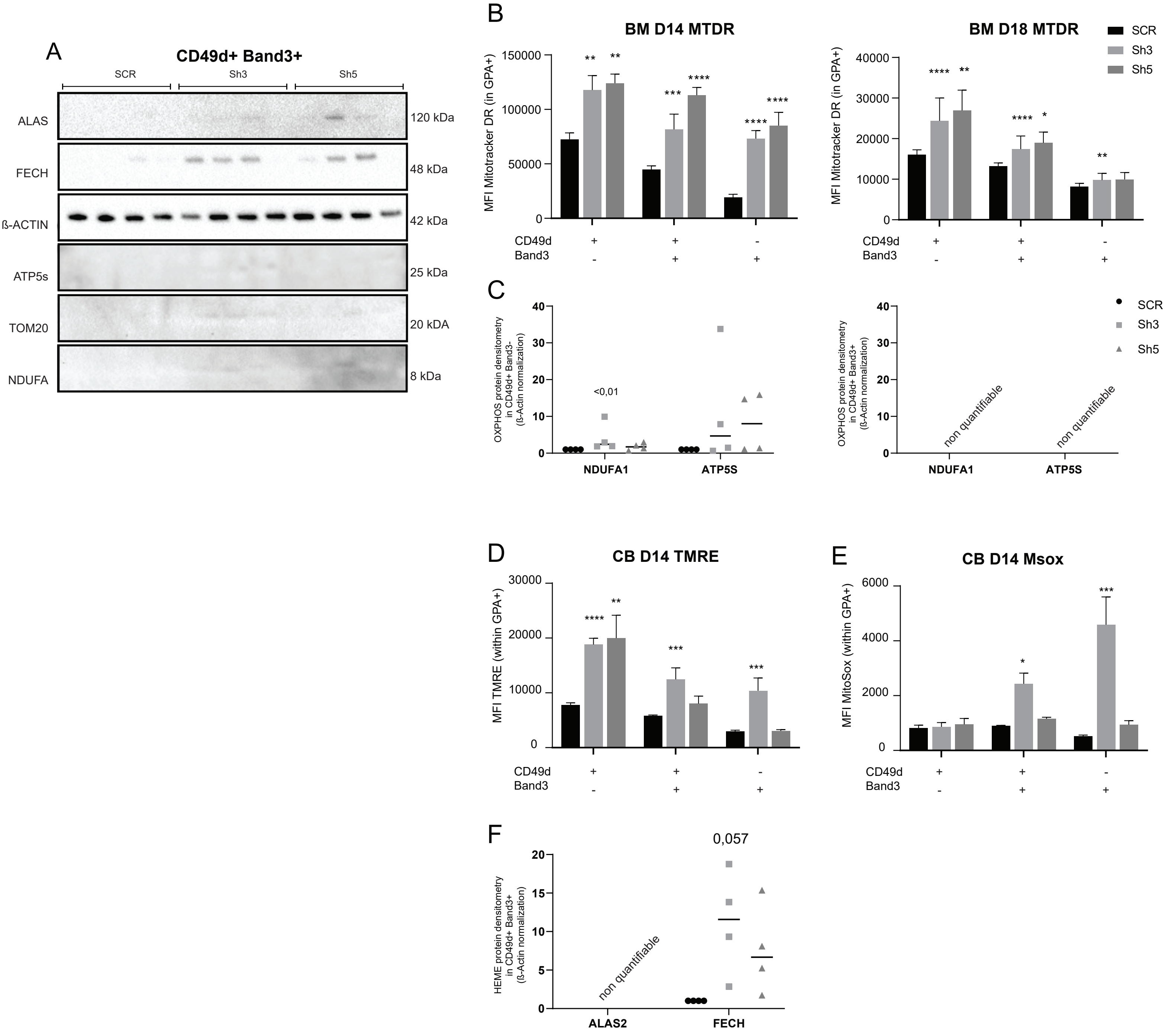

Figure S4. PGC1ß knock-down reults in retained mitochondrial biomass and increased activity on day 14. **A)** Mitochondria related protein levels are close to non-detectable on day 16 , in CB derived orthochromatic erythroblasts, as analazyed by western blot (n=4). **B)** Quantification of mitochondrial biomass using Mitotracker Deep Red at day 14 (left) and 18 (right) in bone marrow derived erythroid progenitors (n= SCR:4, Sh3:12, Sh5:6). **C)** Western blot quantification of OXPHOS related ATP5S and NDUFA1 normalized to ß-ACTIN in polychromatic erythroblasts (left). Bands too faint to quantify protein in orthochromatic erythroblasts (right) , SCR arbitrarily set to "1", data shown as median (n=4). **D)** Quantification of mitochondrial membrane potential using TMRE on day 14 in CB derived erythroid progenitors (n=4). **E)** Quantification of mitochondrial ROS using MitoSox on day 14 in CB derived erythroid progenitors (n=4). **F)** Western blot quantification of heme related ALAS2 and FECH normalized to ß-ACTIN on day 16 in orthochromatic erythroblasts, SCR arbitrarily set to "1", data shown as median (n=4). Data is presented as mean ± SEM (*P ≤ 0.05, **P ≤ 0.01, ***P ≤ 0.001, ****P ≤ 0.0001).

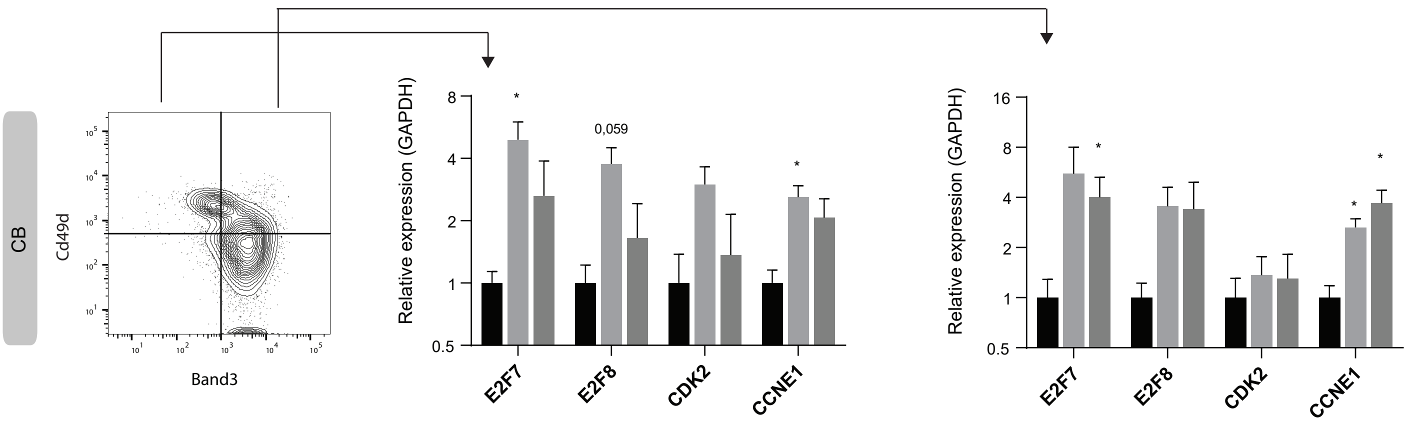

Figure S5. PGC1ß knock-down reults in increased expression of cell cycle regulating genes in CB derived eryhtroid progenitors.
Representative plot of SCR transduced cells indicating populations analyzed for gene expression of cell cycle related genes involved in G1 to S phase progression. Quantification of expression relative to GAPDH on day 16 of polychromatic erythroblasts (left) and orthochromatic erythroblasts (right) (n=4) Data is presented as mean ± SEM (*P ≤ 0.05).

Sen et al Supplemental tables

Supplemental table 1. shRNA's used for the knockdown of PGC1B.

TRC number for specfic shPGC1B clones with corresponding shRNA sequence.

sh#	Clones	Sequence
sh3	TRCN0000008600	CCGGGCATAGTCTAGGCAAAGAAATCTCGAGATTTCTTTGCCTAGACTATGCTTTTT
sh5	TRCN0000429958	CCGGAGCAACTCTATGCTGACTTTCCTCGAGGAAAGTCAGCATAGAGTTGCTTTTTTTG
SCR	Control	CCGGCAACAAGATGAAGAGCACCAACTCGAGTTGGTGCTCTTCATCTTGTTGTTTTT

Supplemental table 2. Primers from IDT and TaqMan, used for RT-qPCR assays.

Included assay names are specific to manufacturer predesigned primers.

Gene	Assay name	Manufacturer
GAPDH	Hs.PT.39a.22214836	IDT
PPARGC1B	Hs.PT.58.38577994	IDT
PPARGC1A	Hs.PT.58.14965839	IDT
COX7B	Hs.PT.58.27185131	IDT
SDHB	Hs.PT.58.39555113	IDT
NDUFA1	Hs.PT.58.20113449	IDT
ATP5S	Hs.PT.58.25982660	IDT
TMEM14C	Hs.PT.58.1926884	IDT
ALAS2	Hs.PT.56a.1433425.g	IDT
ABCB7	Hs.PT.58.2334204	IDT
FECH	Hs.PT.58.25999059	IDT
E2F7	Hs.PT.58.39751342	IDT
E2F8	Hs.PT.58.22500779	IDT
CDK2	Hs.PT.58.302089	IDT
CCNE1	Hs.PT.56a.27776605	IDT
RB1	Hs.PT.58.785814	IDT
HBA	Hs00361191_g1	TaqMan
HBB	Hs00758889_s1	TaqMan

Supplemental Methods

Morphologic scoring DAB stained slides

Cytospin slides were visually scored with Olympus BX43 microscope (Olympus Corporation, Tokyo, Japan), using 40x magnification, into five different developmental stages based on presence and size of nucleus, hemoglobin content and general cell size. Apoptotic cell displaying blebbing and or fragmentation were excluded. 300 cells were counted and scored based on known morphologic characteristics.

Cell size and hemoglobin stain intensity was scored using ImageJ (v1.153c). Scale was set to 8.55pixels/μm using in figure scale bar from ImageView software (v 3.7, YSC Technologies, Fremont CA, USA). Nucleated cells were manually excluded. For quantification of cell size, images were converted to grayscale by changing type to 8-bit and further converted to binary format. Cell size was quantified using ‘Analyze particles’ with a size threshold of 50-150μm^2 and unadjusted circularity (0.00-1.00). At least 20 intact reticulocytes were scored per transduction.

For quantification of hemoglobin content, at least 20 intact reticulocytes were manually selected with multi-point tool per transduction, followed by subtraction of image noise, grayscale conversion and inversion of image. Inversion is done to avoid white background giving highest intensity. Reticulocytes were then analyzed for DAB color intensity by measuring mean grey value.

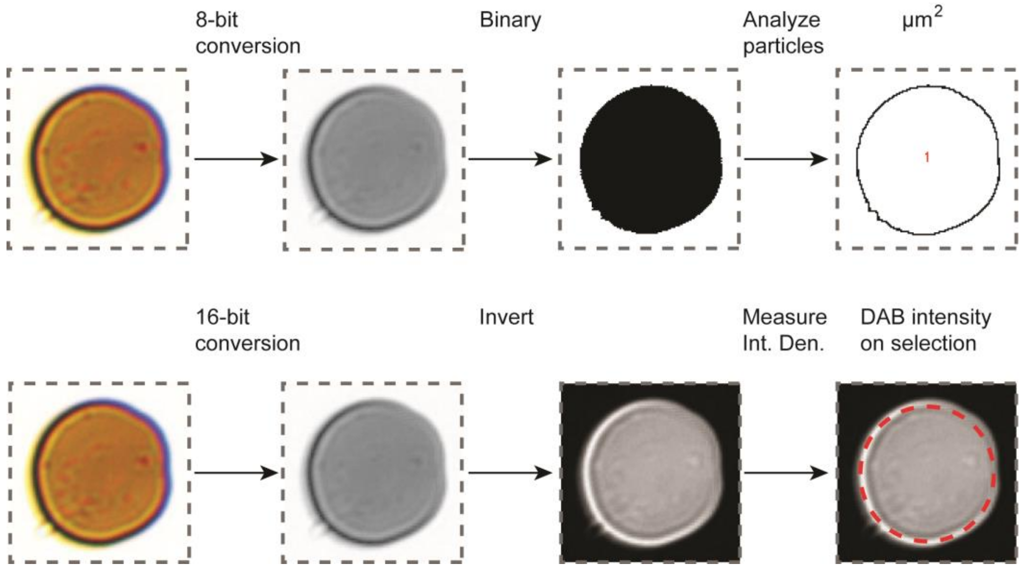

Supplement: Supplementary file 1 — Supplementary Information. [file 41598_2021_96585_MOESM1_ESM.pdf]
